# Detrimental effects of lipopolysaccharides on maturation of bovine oocytes

**DOI:** 10.5713/ajas.18.0540

**Published:** 2018-10-26

**Authors:** Shanjiang Zhao, Yunwei Pang, Xueming Zhao, Weihua Du, Haisheng Hao, Huabin Zhu

**Affiliations:** 1 Embryo Biotechnology and Reproduction Laboratory, Institute of Animal Science, Chinese Academy of Agricultural Sciences, Beijing 100193, China

**Keywords:** Cattle, Lipopolysaccharide, Oocyte, Oxidative Stress, Apoptosis

## Abstract

**Objective:**

Gram-negative bacteria lipopolysaccharide (LPS) has been reported to be associated with uterine impairment, embryonic resorption, ovarian dysfunction, and follicle retardation. Here, we aimed to investigate the toxic effects of LPS on the maturation ability and parthenogenetic developmental competence of bovine oocytes.

**Methods:**

First, we developed an *in vitro* model to study the response of bovine cumulus-oocyte complexes (COCs) to LPS stress. After incubating germinal vesicle COCs in 10 μg/mL of LPS, we analyzed the following three aspects: the expression levels of the LPS receptor toll-like receptor 4 (TLR4) in COCs, activities of intracellular signaling protein p38 mitogen-activated protein kinase (p38 MAPK) and nuclear factor-kappa B (NF-κB); and the concentrations of interleukin (IL)-1β, tumor necrosis factor (TNF)-α, and IL-6. Furthermore, we determined the effects of LPS on the maturation ability and parthenogenetic developmental competence of bovine oocytes.

**Results:**

The results revealed that LPS treatment significantly elevated *TLR4* mRNA and protein expression levels in COCs. Exposure of COCs to LPS also resulted in a marked increase in activity of the intracellular signaling protein p-p38 MAPK and NF-κB. Furthermore, oocytes cultured in maturation medium containing LPS had significantly higher concentrations of the proinflammatory cytokines IL-1β, TNF-α, and IL-6. LPS exposure significantly decreased the first polar body extrusion rate. The cytoplasmic maturation, characterized by polar body extrusion and distribution of peripheral cortical granules, was significantly impaired in LPS-treated oocytes. Moreover, LPS exposure significantly increased intracellular reactive oxygen species levels and the relative mRNA abundance of the antioxidants thioredoxin (*Trx*), *Trx2*, and peroxiredoxin 1 in oocytes. Moreover, the early apoptotic rate and the release of cytochrome *C* were significantly increased in response to LPS. The cleavage, morula, and blastocyst formation rates were significantly lower in parthenogenetically activated oocytes exposed to LPS, while the incidence of apoptotic nuclei in blastocysts was significantly increased.

**Conclusion:**

Together, these results provide an underlying mechanism by which LPS impairs maturation potential in bovine oocytes.

## INTRODUCTION

Lipopolysaccharide (LPS) is a toxic component occurring in the cell walls of gram-negative bacteria and has been found to cause delayed ovulation and reduced fecundity in cattle [1,2]. In general, bacterial infections of the uterus are ubiquitous in human and animals, including cattle, after coitus and/or parturition [3], and up to 20% of animals failed to achieve pregnancy after infection of the endometrium with *Escherichia coli* [4,5]. Many studies have reported that LPS accumulation in the follicular fluid of animals with metritis may cause ovarian dysfunction, aberrant changes in the oviductal transcriptome profile, and perturbation of follicular development [6–8].

LPS is known to initiate several destructive processes via interaction with toll-like receptor 4 (TLR4), which is a member of the TLR family of transmembrane proteins [9,10]. TLR4 binds to LPS, triggering a cascade of signal transduction events that evoke the release of numerous biochemical mediators such as cytokines and toxic free radicals [8,11]. Blum et al [12] studied dairy cows and found that LPS could induce tumor necrosis factor (TNF)-α and Nox (nitrite and nitrate) accumulation in milk and plasma. Bovine and murine granulosa cells express TLR4 receptor complex, respond rapidly to LPS with phosphorylation of the TLR signaling components p38 and extracellular signal-regulated kinase (ERK), and increase the abundance of interleukin (IL) 6 and IL8 transcripts [3,13]. In addition, LPS has been reported to directly affect the intracellular redox status and induce apoptotic incidence by enhancing the release of pro-apoptotic factors [14]. Thus, we hypothesize that LPS-induced immune challenges in bovine cumulus-oocyte complexes (COCs) have a deleterious effect on both the maturation and developmental competence of follicle-enclosed oocytes.

Mammalian oocyte maturation involves nuclear and cytoplasmic maturation, which are essential for successful fertilization and embryonic development [15]. The developmental competence of oocytes depends on follicular development and includes a variety of molecular and cellular events [16]. Higher and moderate LPS levels have been detected in the follicular fluid of growing follicles collected from postpartum cows with endometritis as well as from women with bacterial vaginosis [3,17,18]. Ibrahim et al [8] found that the viability of oviduct epithelial cells was significantly decreased following LPS challenge. In the presence of LPS, oocytes failed to reach metaphase II (MII) or exhibited aberrant meiotic structures. However, the exact mechanisms by which LPS affects oocyte competence during *in vitro* maturation (IVM) remains unclear. Thus, in the present study we aimed to assess the detrimental effect of LPS on oocyte maturation potential, and to investigate the mechanisms involved in this process.

## MATERIALS AND METHODS

### Antibodies and chemicals

Unless otherwise specified, all chemicals were purchased from Sigma-Aldrich Co. (St. Louis, MO, USA). Rabbit polyclonal antibodies to nuclear factor-kappa B (NF-κB) and TLR4 were from Novus (Novus, Littleton, CO, USA). Rabbit polyclonal antibodies to cytochrome *C* were obtained from Abcam (Cambridge, UK). p-p38 mitogen-activated protein kinase (p38 MAPK) and p-p38 MAPK were purchased from Cell Signaling Technology (Beverly, MA, USA). Alexa Fluor-488-conjugated mouse anti-rabbit secondary antibodies were purchased from Invitrogen (Carlsbad, CA, USA). Annexin V-FITC kits were purchased from Vazyme Biotech Co., Ltd. (Nanjing, China). *In Situ* Cell Death Detection Kit was obtained from Roche (Mannheim, Germany). All experimental procedures were conducted according to the guidelines of the Institutional Animal Care and Use Committee at the Chinese Academy of Agricultural Sciences.

### Oocyte harvest and *in vitro* maturation

Bovine ovaries were transported from the local abattoirs to the laboratory in sterile physiological saline solution, and maintained at 26°C to 30°C within 2 h of harvesting. Before ovaries were aspirated, they were washed three times with warm physiological saline. The COCs that were 2 to 6 mm in diameter were manually aspirated from antral follicles using an 18-gauge needle. Oocytes surrounded by at least three compact cumulus layers and had uniform ooplasm were selected for the next step. Generally, prior to IVM, COCs should be washed thrice in IVM medium. Then, 50 COCs were incubated in 750 μL of maturation medium in each well of a four-well plate (Nunc, Denmark) in air containing 5% CO_2_ at 38.5°C and maximum humidity. The IVM was carried out in TCM-199 (Gibco BRL, Grand Island, NY, USA) containing 0.01 IU/mL follicle-stimulating hormone, 10 IU/mL luteinizing hormone, 1 μg/mL estradiol, 10 mg/mL heparin, and 10% (v/v) fetal bovine serum (FBS; Gibco BRL, USA).

### Lipopolysaccharide treatment

LPS was dissolved in water and diluted with IVM medium to obtain a final concentrations of 10 μg/mL. The proportion of LPS in the final culture medium should not exceed 1% after dilution with IVM medium.

### Parthenogenetic activation and *in vitro* culture

Oocytes were harvested from bovine ovaries and pretreated with 10 μg/mL LPS for 22 h. They were then subjected to parthenogenetic activation and *in vitro* embryo culture in the absence of LPS until they reached the blastocyst stage. Oocytes were treated with 5 μM Ca^2+^ ionophore A23187 for 5 min and incubated in 2 mM of 6-dimethylaminopurine for another 6 h. Following activation, oocytes were cultured in Charles Rosenkrans medium with added amino acid (CR1aa) supplemented with 0.1% (w/v) bovine serum albumin (BSA) for 48 h at 38.5°C in air containing 5% CO_2_. They were then cultured for an additional 5 days in CR1aa-supplemented medium with 10% (v/v) FBS. The medium was changed every two days throughout the culture period. Cleavage, morula, and blastocyst formation rates were recorded at 2, 6, and 7 days, respectively. Each test was repeated three times, and 30 oocytes were analyzed per group in each replicate.

### Cell counting

For cell counting, blastocysts were collected on day 7 and stained as previously described [19]. All blastocysts were washed with phosphate-buffered saline (PBS) containing 1 mg/mL polyvinyl alcohol (PVA), and fixed in 500 μL ethanol containing 10 μg/mL Hoechst-33342 for 30 min. After rinsing in 0.1% PVA/PBS, the stained blastocysts were mounted onto slides and analyzed under a confocal microscope (Olympus FV1000, Tokyo, Japan). Each test was repeated three times, and 30 oocytes were analyzed per group in each replicate.

### Cytochemical staining

The COCs were cultured for 22 h, after which cumulus cells were removed by repeated pipetting. Denuded oocytes were fixed in 3.7% (w/v) paraformaldehyde in PBS for 1 h, and permeabilized with PBS supplemented with 1% Triton X-100 for 1 h. After the cells were washed three times in PBS containing 0.1% PVA, the oocytes were blocked in 1% BSA/PBS for 8 h. The oocytes were incubated with antibodies against TLR4 (1:500), NF-κB (1:500), and cytochrome *C* (1:1,000) overnight at 4°C. After washing three times (5 min each time) in PBS-0.1% PVA medium, the oocytes were labeled with secondary antibodies for 40 min at room temperature and then incubated with 2 μg/mL 4′,6-diamidino-2-phenylindole (Roche, Germany). Samples from each group were transferred onto identical slides and analyzed under a confocal microscope (Olympus FV1000, Japan).

To study the distribution of peripheral cortical granules (CGs) and to determine the cellular levels of reactive oxygen species (ROS), the samples were stained with 10 μg/mL of fluorescein isothiocyanate (FITC)-labeled peanut agglutinin and 10 μM 2′,7′-dichlorodihydrofluorescein diacetate in the dark for 30 min, and then washed thrice in washing buffer. Then, apoptosis was detected using an *In Situ* Cell Death Detection Kit and an Annexin V/PI-FITC kit. Samples from each group were transferred onto identical slides and analyzed under a confocal microscope (Olympus FV1000, Japan).

Each test was repeated three times, and at least 15 oocytes were analyzed per group in each replicate. The fluorescence intensity was analyzed using ImageJ software (National Institutes of Health, Bethesda, MD, USA), and the fluorescence intensity per pixel within the images was analyzed using the ROI (National Institutes of Health, USA). Finally, the average values of all images were calculated and used as the final intensities for each group.

### Enzyme-linked immunosorbent assay

IL-1β, IL-6, and TNF-α were measured from cell-free supernatants using commercially available bovine-specific enzyme-linked immunosorbent assay kits (LBTR, Beijing, China) according to the manufacturer’s instructions. Each test was repeated three times.

### Real-time quantitative polymerase chain reaction

The total RNA was extracted from 100 oocytes with or without LPS treatment using Trizol reagent (Invitrogen, USA) for 22 h. First-strand cDNA was synthesized from 0.05 μg of RNA using random hexamers. Quantitative real-time polymerase chain reaction was conducted in three replicates using an ABI-7900 SDS instrument (Applied Biosystems, Foster city, CA, USA), and the primers used are listed in Table 1. The expression level of the genes was calculated using the 2^−ΔΔc(t)^ method, and glyceraldehyde-3-phosphate dehydrogenase was used as the reference gene. Each test was repeated three times, and 100 oocytes were analyzed per group in each replicate.

### Western blot analysis

For the Western blot analysis, 150 bovine oocytes at the MII stage were harvested and washed thrice in PBS. The proteins were extracted and seperated by sodium dodecyl sulfate-polyacrylamide gel electrophoresis using 7.5% polyacrylamide gel and tranferred onto membranes (Millipore Corp, Billerica, MA, USA). The membranes were then blocked in 5% (w/v) skim milk overnight at 4°C, and incubated with p-p38 MAPK monoclonal antibodies (1:1,000 dilution) and p38 MAPK antibodies (1:1,000) at 4°C overnight, washed thrice in TBS for five minutes each time, and incubated with horseradish peroxidase-conjugated secondary antibodies (Zhong Shan Biotechnology, Beijing, China) for 1 h at room temperature. The signals were detected using an ECL kit (Tanon, Shanghai, China). Each test was repeated three times, and 150 oocytes were analyzed per group in each replicate.

### Statistical analysis

At least three biological replicates were used, and the results were presented as means±stsandard error of the mean. Data were statistically analyzed using the *t* test. Less than 0.05 p value was considered statistically significant.

## RESULTS

### Lipopolysaccharide exposure induces inflammation in bovine cumulus-oocyte complexes

**Figure 1:**
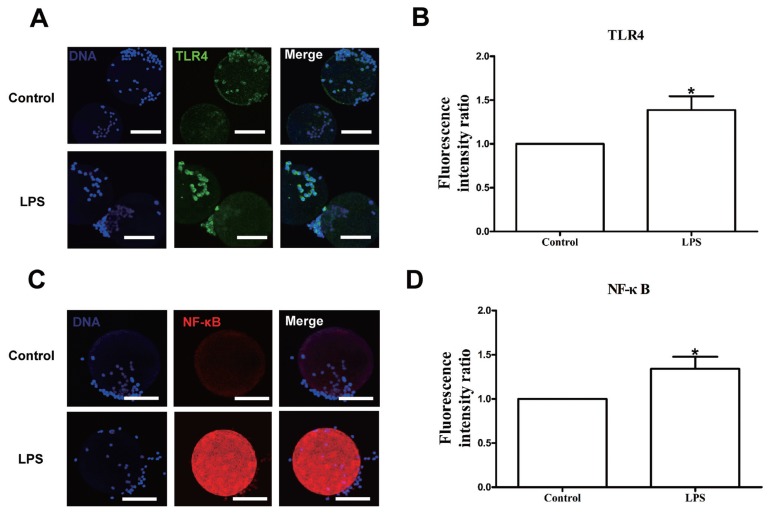
Lipopolysaccharide exposure increases the TLR4 and NF-κB levels in bovine cumulus-oocyte complexes (COCs). (A) Immunofluorescence staining for TLR4 in bovine COCs. Blue, chromatin; green, TLR4. Bar = 50 μm. (B) TLR4 levels in bovine COCs. (C) Immunofluorescence staining for NF-κB in bovine COCs. Blue, chromatin; red, NF-κB. Bar = 50 μm. (D) NF-κB levels in bovine COCs. TLR4, toll-like receptor 4; NF-κB, nuclear factor-kappa B; COCs, cumulus-oocyte complexes. Asterisk indicates significant difference (p<0.05).

**Figure 2:**
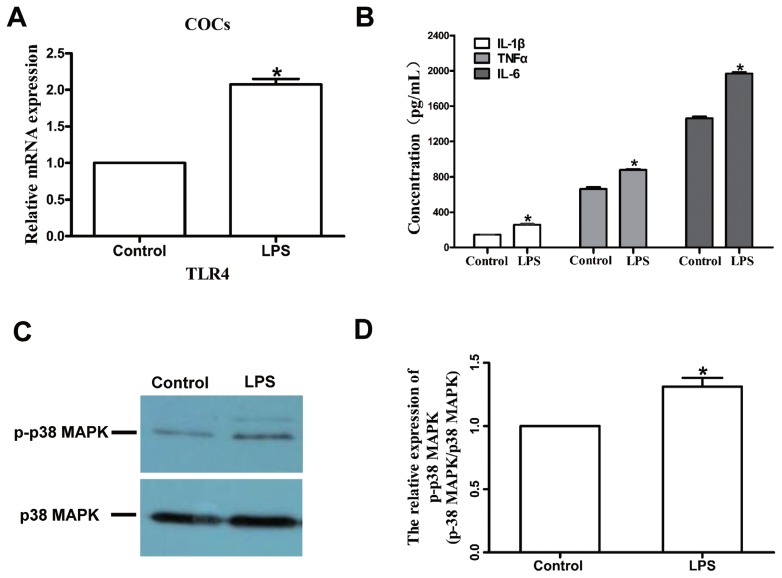
LPS exposure increases the *TLR4* mRNA level, p-p38 MAPK protein level, and production of the proinflammatory cytokines IL-1β, TNF-α, and IL-6. (A) *TLR4* transcript levels in bovine COCs. (B) Production of IL-1β, TNF-α, and IL-6 after exposure to LPS. (C) and (D) Western blot analysis of p-p38 MAPK and p-38 protein levels. LPS, lipopolysaccharide; *TLR4*, toll-like receptor 4; p-p38 MAPK, phosphorylation of p38 mitogen-activated protein kinase; IL, interleukin; TNF-α, tumor necrosis factor-α; COCs, cumulus-oocyte complexes. Asterisk indicates significant difference (p<0.05).

We first measured the expression of TLR4 in bovine COCs. The TLR4 is one of the pattern recognition receptors for LPS, which activates the downstream factors p38 MAPK and NF-κB. Excitation of NF-κB results in the generation of cytokines and other inflammatory mediators such as TNF-α, IL-1β, and IL-6. The *TLR4* mRNA level was upregulated in the LPS group compared with the control group (p<0.05; Figure 2A), and TLR4 and NF-κB protein expression levels were also significantly increased in the LPS group relative to the control (Figure 1). We also measured the concentrations of three proinflammatory cytokines, IL-1β, TNF-α, and IL-6 in cell-free supernatants, and the results revealed that IL-1β, TNF-α, and IL-6 concentrations were significantly higher in the LPS group than in the control group (p<0.05; Figure 2B).

To further confirm the effects of LPS on maturation potential, we analyzed the level of p38 MAPK, which is a typical inflammatory gene expression regulator. As shown in Figure 2C and 2D, the p-p38 MAPK level was significantly higher in the LPS-treated oocytes than in the control oocytes (p<0.05). Collectively, these results indicate that LPS challenge induces an inflammatory response in bovine COCs, which may in turn lead to the low maturation rate of oocytes.

### Lipopolysaccharide exposure inhibits the maturation potential of bovine oocytes

**Figure 3:**
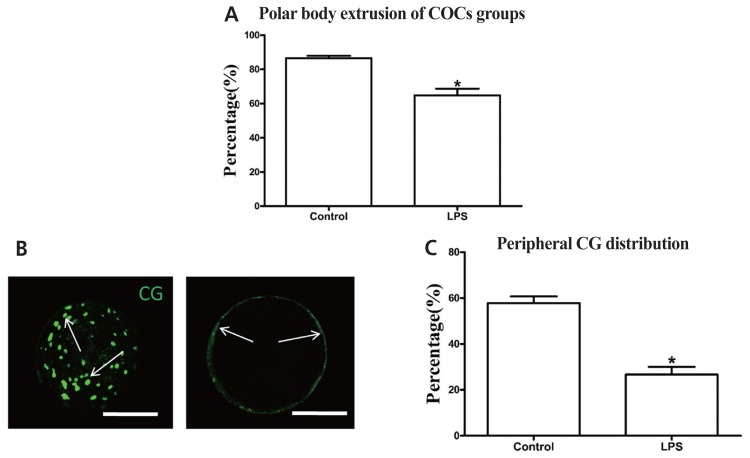
LPS exposure inhibits the maturation potential of bovine oocytes. (A) Polar body extrusion rate after LPS treatment. (B) Representative photomicrographs. Peripheral CGs are indicated with arrows. Green, CG. Bar = 50 μm. (C) Proportion of oocytes with peripheral CG distribution after LPS treatment. LPS, lipopolysaccharide; CGs, cortical granules. Asterisk indicates significant difference (p<0.05).

Exposing oocytes to LPS significantly decreased the first polar body extrusion rate (64.8%±3.89%, n = 402) compared with the control group (86.5%±1.36%, n = 386) (p<0.05; Figure 3A). The proportion of oocytes with peripheral CG distribution (26.7%±3.33%, n = 80) was also significantly lower in the LPS-treated group than in the control group (57.8%±2.94%, n = 80) (p<0.05; Figure 3B, 3C). These results indicate that LPS exposure directly inhibits the maturation potential of bovine oocytes.

### Lipopolysaccharide exposure induces oxidative stress and apoptosis in bovine oocytes

**Figure 4:**
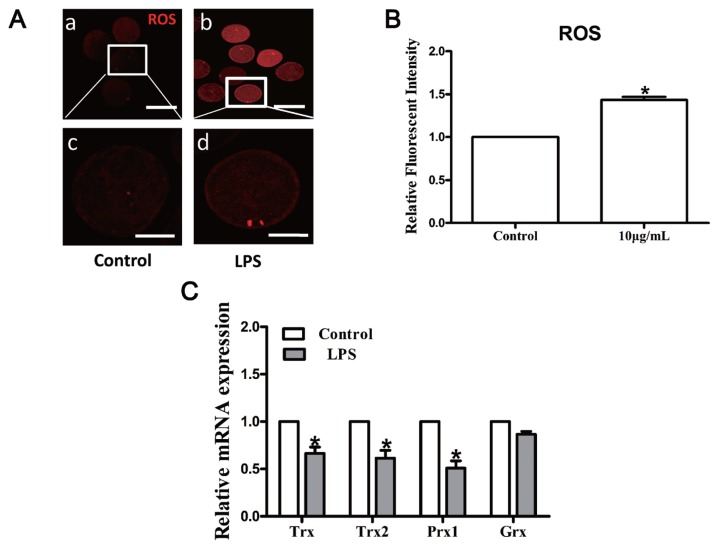
LPS treatment alters intracellular redox state in bovine oocytes. (A) Representative photomicrographs. Red, ROS. a, b: Bar = 100 μm; c, d: Bar = 50 μm. (B) ROS expression after LPS treatment. (C) Transcripts of redox signaling-associated genes *Trx*, *Trx2*, *Prx1*, and *Grx*. LPS, lipopolysaccharide; ROS, reactive oxygen species; *Trx*, thioredoxin; *Prx1*, peroxiredoxin 1; *Grx*, glutaredoxin. Asterisk indicates significant difference (p<0.05).

LPS treatment significantly increased the intracellular ROS levels in MII oocytes compared to the control group (p<0.05; Figure 4A, 4B). The mRNA transcripts of thioredoxin (*Trx*), *Trx2*, and peroxiredoxin 1 (*Prx1*) were markedly decreased following LPS treatment, while glutaredoxin (*Grx*) mRNA level was unaffected (p>0.05; Figure 4C), illustrating that LPS treatment induces oxidative stress in bovine oocytes.

**Figure 5:**
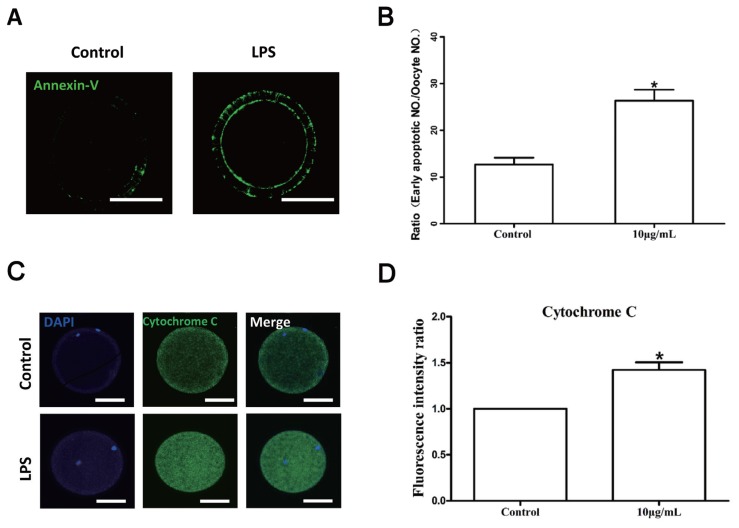
LPS exposure induces apoptosis in bovine oocytes. (A) Representative photomicrographs. Green, Annexin V. Bar = 50 μm. (B) Rates of early apoptosis after LPS treatment. (C) Representative photomicrographs. Green, cytochrome *C*. Bar = 50 μm. (D) Cytochrome C levels in bovine oocytes. LPS, lipopolysaccharide. Asterisk indicates significant difference (p<0.05).

The results of the Annexin V assay revealed a significantly higher early apoptosis rate in oocytes exposed to LPS (26.33% ±2.33%, n = 72) than in untreated oocytes in the control group (12.67%±1.45%, n = 66) (p<0.05; Figure 5A, 5B). Furthermore, the fluorescence signal of cytochrome *C* was also significantly enhanced in the cytoplasm of LPS-treated oocytes (n = 80; p< 0.05, Figure 5C, 5D). These data suggest that the detrimental effect of LPS on oocyte maturation potential may be partly due to its pro-apoptotic activity.

### Lipopolysaccharide exposure decreases parthenogenetic development of bovine oocytes

**Figure 6:**
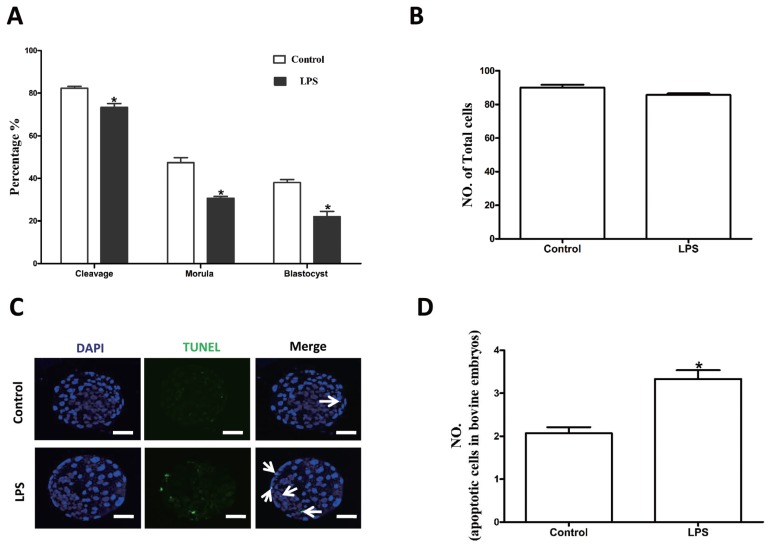
LPS exposure decreases the parthenogenetic development of bovine oocytes. (A) Rates of different stages of parthenogenetic development after oocytes were preincubated with LPS for 22 h during *in vitro* maturation. (B) Total number of cells in blastocysts from each treatment group. (C) Representative photomicrographs. Blue, chromatin; Cyan, the transferase-mediated dUTP nick end labeling (TUNEL)-positive apoptotic nuclei. Bar = 20 μm. LPS, lipopolysaccharide. (D) Apoptotic cell rate. Asterisk indicates significant difference (p<0.05).

We further analyzed the effects of LPS on the parthenogenetic development of bovine oocytes. The cleavage (73.3%±1.76%, n = 230), morula (30.7%±0.88%), and blastocyst formation (14.8%±1.96%) rates were significantly lower in the LPS group than in the control group (82.3%±0.88%, n = 248; 47.3%±2.40%; and 34.2%±0.8%, respectively) (p<0.05; Figure 6A). The average number of total cells in blastocysts did not significantly differ between the control (89.3%±1.75%, n = 30) and treatment (85.2%±2.05%, n = 30) groups (p>0.05; Figure 2B). However, LPS treatment significantly increased the incidence of apoptotic nuclei in parthenogenetic blastocysts (3.33±0.20, n = 32) compared to the control group (2.06±0.15, n = 32) (p<0.05; Figure 6C, 6D). Together, these data imply that exposure of bovine oocytes to LPS during IVM affected their quality and reduced their ability to subsequently undergo parthenogenetic development.

## DISCUSSION

We first developed an *in vitro* model of LPS stress in bovine COCs. The current study was conducted to test the effects of LPS on the maturation ability and parthenogenetic developmental competence of bovine oocytes. The results demonstrated that LPS exposure significantly decreased the maturation rate of bovine oocytes and induced oxidative stress and apoptosis during the maturation of bovine oocytes. In addition, the development and quality of bovine parthenotes produced *in vitro* and derived from LPS-treated oocytes were obviously impaired. During ovulation, COCs generate a physical matrix around the oocyte; in addition, cumulus and granulosa cells also express considerable immune cell-like function [20]. It is well established that TLRs, which can recognize pathogen-associated molecules such as LPS, play important roles in initiating inflammatory responses orchestrated by proinflammatory cytokines such as IL-1, IL-6, and TNF-α [21]. In cows, granulosa cells trigger an immune response to LPS via the TLR4 pathway, leading to inflammation and perturbation of meiotic competence [13]. COCs exposed to LPS exhibit reinforced phosphorylation levels of p38 MAPK, increased ERK1/2 and NF-κB activity, and increased IL-6 and TNF-α expression levels. As phosphorylation of p38 MAPK and induction of IL-6 and TNF-α expression are downstream events initiated by activation of the TLR pathway, Shimada et al [20] proposed that the TLR pathway may play critical roles during the ovulation process. In the current study, exposure of COCs to LPS upregulated the *TLR4* mRNA and protein levels, and increased the concentrations of IL-1β, TNF-α, and IL-6 in the supernatant in the presence of LPS. Furthermore, p38 MAPK and NF-κB pathways were activated in response to LPS. We speculated that LPS could trigger inflammatory processes in COCs via the TLR4 pathway, and then affect oocyte maturation.

Previously, it has been demonstrated that the maturation and developmental competence of oocytes could be damaged in a bacterial-type-dependent manner [22]. Oocytes treated with gram-negative endotoxin (i.e., LPS) exhibited impaired nuclear and cytoplasmic maturation, characterized by a reduced proportion of MII oocytes and CGs distributed in the cortical region [23]. The number of oocytes that failed to reach the MII stage of meiosis or those with perturbed meiotic structures such as aberrant spindles, chromosomal ejection, and germinal vesicle breakdown failure was significantly increased following exposure to LPS [13]. Consistent with the above results, our study further confirmed that LPS exposure inhibited nuclear and cytoplasmic maturation of bovine oocytes. LPS is primarily recognized as a proinflammatory and immunomodulatory molecule [24]. However, many studies have demonstrated that LPS could release superoxide radicals and other ROS, leading to oxidative stress and programmed cell death in different cell lines [14,25,26]. As expected, LPS exposure significantly increased the intracellular ROS levels and significantly decreased the transcript abundance of detoxification enzymes including *Trx*, *Trx2*, and *Prx1* in MII bovine oocytes. Additionally, LPS-challenged oocytes exhibited significant increases in the early apoptotic rate and released cytochrome *C*. In a recent study on human gingival fibroblasts, LPS was found to markedly elevate mitochondrial and cytoplasmic ROS levels [27]. Similarly, another study found elevated apoptotic cell rate and cytosolic cytochrome *C* and caspase-3 activities in rat alveolar epithelial cells *in vivo* and *in vitro* after challenge with LPS [28]. The similar results obtained in this study suggest that the detrimental effects of LPS on oocyte maturation potential may be partly via the induction of oxidative stress and apoptosis.

Given the prominent effect of LPS on oocyte maturation, we further evaluated the effect of LPS on the embryonic development and quality of LPS-treated parthenogenetically activated oocytes. The cleavage, morula, and blastocyst formation rates showed obvious decreases in the oocytes that had been exposed to LPS. LPS significantly increased the apoptotic signals but not the total number of nuclei in embryos. LPS has been associated with adverse developmental outcomes such as embryonic resorption, fetal death, and growth retardation [29]. In a previous study, oocytes that had been matured in follicular fluid aspirated from LPS-induced mastitic cows showed significantly impaired developmental competence [23]. Ibrahim et al [8] found that LPS could adversely affect the proportion of embryos that develop to the blastocyst stage. Our results are consistent with reports from previous literature and suggest that exposure of oocytes to environments exposed to LPS could reduce their developmental potential.

## CONCLUSION

In conclusion, our findings demonstrate that LPS could reduce the maturation ability and parthenogenetic developmental competence of bovine oocytes *in vitro*. Our findings suggest that the immune response, oxidative stress, and pro-apoptotic effects of LPS may be the potential mechanisms underlying this effect.

## Figures and Tables

**Table 1:** Primer sequences used for real-time quantitative polymerase chain reaction

Gene	Gene ID	Primer	Sequence (5′3′)	Annealing temperature (°C)	Fragment size (bp)
*GAPDH*	281181	Forward	GGGTCATCATCTCTGCACCT	60	177
		Reverse	GGTCATAAGTCCCTCCACGA		
*TLR4*	281536	Forward	CTTGCGTACAGGTTGTTCCTAA	56	153
		Reverse	CTGGGAAGCTGGAGAAGTTATG		
*Trx*	280950	Forward	TGGTGTTCCTTGAAGTAGATGTGG	60	109
		Reverse	TCACCCACCTTCTGTCCCTTT		
*Trx2*	281557	Forward	GTTCCTGACCTCCATAATCTCCG	61	121
		Reverse	GGCTTGGCTGGGTGTTACTGT		
*Prx1*	281997	Forward	CGCTGGTGTCGGTCCTATTT	60	98
		Reverse	GCTGTTGCTTTGAACTGGGG		
*Grx*	515416	Forward	TACCTCGGGTCTTCATCGGT	55	103
		Reverse	GCTCCCATTTGCTTTAGCCG		

*GAPDH*, glyceraldehyde-3-phosphate dehydrogenase; *TLR4*, toll-like receptor 4; *Trx*, thioredoxin; *Prx1*, peroxiredoxin 1; *Grx*, glutaredoxin.

## References

[1] Sheldon IM, Noakes DE, Rycroft AN, Pfeiffer DU, Dobson H (2002). Influence of uterine bacterial contamination after parturition on ovarian dominant follicle selection and follicle growth and function in cattle. Reproduction.

[2] Williams EJ, Sibley K, Miller AN (2008). The effect of *Escherichia coli* lipopolysaccharide and tumour necrosis factor alpha on ovarian function. Am J Reprod Immunol.

[3] Herath S, Williams EJ, Lilly ST (2007). Ovarian follicular cells have innate immune capabilities that modulate their endocrine function. Reproduction.

[4] Borsberry S, Dobson H (1989). Periparturient diseases and their effect on reproductive performance in five dairy herds. Vet Rec.

[5] LeBlanc SJ, Duffield TF, Leslie KE (2002). Defining and diagnosing postpartum clinical endometritis and its impact on reproductive performance in dairy cows. J Dairy Sci.

[6] Karsch FJ, Battaglia DF, Breen KM, Debus N, Harris TG (2002). Mechanisms for ovarian cycle disruption by immune/inflammatory stress. Stress.

[7] Sheldon IM, Cronin J, Goetze L, Donofrio G, Schuberth HJ (2009). Defining postpartum uterine disease and the mechanisms of infection and immunity in the female reproductive tract in cattle. Biol Reprod.

[8] Ibrahim S, Salilew-Wondim D, Rings F (2015). Expression pattern of inflammatory response genes and their regulatory micrornas in bovine oviductal cells in response to lipopolysaccharide: implication for early embryonic development. PLoS One.

[9] Raetz CR, Whitfield C (2002). Lipopolysaccharide endotoxins. Annu Rev Biochem.

[10] Beutler B (2004). Inferences, questions and possibilities in Toll-like receptor signalling. Nature.

[11] Doyle SL, O’Neill LA (2006). Toll-like receptors: from the discovery of NFkappaB to new insights into transcriptional regulations in innate immunity. Biochem Pharmacol.

[12] Blum JW, Dosogne H, Hoeben D (2000). Tumor necrosis factor-alpha and nitrite/nitrate responses during acute mastitis induced by *Escherichia coli* infection and endotoxin in dairy cows. Domest Anim Endocrinol.

[13] Bromfield JJ, Sheldon IM (2011). Lipopolysaccharide initiates inflammation in bovine granulosa cells via the TLR4 pathway and perturbs oocyte meiotic progression *in vitro*. Endocrinology.

[14] Raza H, John A, Shafarin J (2016). Potentiation of LPS-induced apoptotic cell death in human hepatoma HepG2 cells by aspirin via ROS and mitochondrial dysfunction: protection by N-acetyl cysteine. PLoS One.

[15] Coticchio G, Dal Canto M, Mignini Renzini M (2015). Oocyte maturation: gamete-somatic cells interactions, meiotic resumption, cytoskeletal dynamics and cytoplasmic reorganization. Hum Reprod Update.

[16] Coticchio G, Sereni E, Serrao L, Mazzone S, Iadarola I, Borini A (2004). What criteria for the definition of oocyte quality?. Ann NY Acad Sci.

[17] Platz-Christensen JJ, Mattsby-Baltzer I, Thomsen P, Wiqvist N (1993). Endotoxin and interleukin-1 alpha in the cervical mucus and vaginal fluid of pregnant women with bacterial vaginosis. Am J Obstet Gynecol.

[18] Bannerman DD, Paape MJ, Hare WR, Sohn EJ (2003). Increased levels of LPS-binding protein in bovine blood and milk following bacterial lipopolysaccharide challenge. J Dairy Sci.

[19] Thouas GA, Korfiatis NA, French AJ, Jones GM, Trounson AO (2001). Simplified technique for differential staining of inner cell mass and trophectoderm cells of mouse and bovine blastocysts. Reprod Biomed Online.

[20] Shimada M, Hernandez-Gonzalez I, Gonzalez-Robanya I, Richards JS (2006). Induced expression of pattern recognition receptors in cumulus oocyte complexes: novel evidence for innate immune-like functions during ovulation. Mol Endocrinol.

[21] Takeuchi O, Akira S (2010). Pattern recognition receptors and inflammation. Cell.

[22] Roth Z, Asaf S, Furman O (2015). Subclinical mastitis disrupts oocyte cytoplasmic maturation in association with reduced developmental competence and impaired gene expression in preimplantation bovine embryos. Reprod Fertil Dev.

[23] Asaf S, Leitner G, Furman O (2014). Effects of Escherichia coli- and Staphylococcus aureus-induced mastitis in lactating cows on oocyte developmental competence. Reproduction.

[24] Jaja-Chimedza A, Gantar M, Mayer GD, Gibbs PD, Berry JP (2012). Effects of cyanobacterial lipopolysaccharides from *microcystis* on glutathione-based detoxification pathways in the zebrafish (*Danio rerio*) embryo. Toxins (Basel).

[25] Sagar S, Kumar P, Behera RR, Pal A (2014). Effects of CEES and LPS synergistically stimulate oxidative stress inactivates OGG1 signaling in macrophage cells. J Hazard Mater.

[26] Qin T, Yin Y, Yu Q, Yang Q (2015). Bursopentin (BP5) protects dendritic cells from lipopolysaccharide-induced oxidative stress for immunosuppression. PLoS One.

[27] Li X, Wang X, Zheng M, Luan QX (2016). Mitochondrial reactive oxygen species mediate the lipopolysaccharide-induced pro-inflammatory response in human gingival fibroblasts. Exp Cell Res.

[28] Fu C, Dai X, Yang Y, Lin M, Cai Y, Cai S (2017). Dexmedetomidine attenuates lipopolysaccharide-induced acute lung injury by inhibiting oxidative stress, mitochondrial dysfunction and apoptosis in rats. Mol Med Rep.

[29] Zhao L, Chen YH, Wang H (2008). Reactive oxygen species contribute to lipopolysaccharide-induced teratogenesis in mice. Toxicol Sci.

